# Supplementary material for: Developing a decision instrument to guide abdominal-pelvic imaging of blunt trauma patients: Methodology and protocol of the NEXUS abdominal-pelvic imaging study
Source: PLoS One. 2022 Jul 25;17(7):e0271070. doi: 10.1371/journal.pone.0271070 (PMC9312398; doi:10.1371/journal.pone.0271070)
Supplement: S1 File — (DOCX) [file pone.0271070.s001.docx]

| **Supplement 1**  **Abdominal/Pelvic CT Clinical Questionnaire** | | | | | | | | | | | | | | | | | | | | | | | | | | |
| --- | --- | --- | --- | --- | --- | --- | --- | --- | --- | --- | --- | --- | --- | --- | --- | --- | --- | --- | --- | --- | --- | --- | --- | --- | --- | --- |
| **Demographics/Clinical Presentation** | | | | | | | | | | | | | | | | | | | | | | | | | | |
| **Demographics** | | | | | | | | | | | | | | | | | | | | | | | | | | |
| **Date of Birth** | ____/____/_______ *mm/dd/yyyy* | | | | | | | | **Gender** | | | | Male | | | | Female | | |  | | | | | | |
| **Race** | White | | Black | | | Asian | | | Native American | | | | | | | | Middle-Eastern | | | | | Other | | | Unknown | |
| **Ethnicity** | Hispanic | | | Non-Hispanic | | | | |  | | | | | | | | | | | | | | | | | |
| **cLINICAL PRESENTATION** | | | | | | | | | | | | | | | | | | | | | | | | | | |
| **Patient Status** | Stable | | | | Unstable | | | | | **Indication for CT** | | | | | | Trauma | | | | | Other | | | | | |
| **Coagulopathy or Anticoagulant Use** | | | | | Yes | | | No | | Unknown | | | | | | Specific agents(s): ______________________________________ | | | | | | | | | | |
| **Rapid Deceleration Mechanism** | | | | | Yes | | | No | | Unknown | | | | | | Specific: ______________________________________________ | | | | | | | | | | |
|  | | | | | | | | | | | | | | | | | | | | | | | | | | |
| **EVALUATION** | | | | | | | | | | | | | | | | | | | | | | | | | | |
| **Initial O_2_ saturation (%)** _____ | | | | | | | **Initial Pulse Rate (BPM)** ______ | | | | | | | | | | | **Initial Systolic Blood Pressure (mm Hg)** ______ | | | | | | | | |
| **Lowest ED O_2_ saturation (%)** _____ | | | | | | | **Highest ED Pulse Rate (BPM)** ______ | | | | | | | | | | | **Lowest ED Systolic Blood Pressure (mm Hg)** ______ | | | | | | | | |
| **Initial Hemoglobin (g/dl)** ______  Not Available | | | | | | | | | | | | **Initial Serum Lactate (mg/dl)** ______  Not Available | | | | | | | | | | | | | | |
| Please indicate whether each of the following characteristics are present, ABSENT, OR CANNOT BE ASSESSED: | | | | | | | | | | | | | | | | | | | | | | | | | | |
| **Abdominal Pain or Tenderness** | | | | | | | | | | | | | | | | | | Yes | | | | | No | | Unknown | |
| **Flank Pain or Tenderness** | | | | | | | | | | | | | | | | | | Yes | | | | | No | | Unknown | |
| **Pelvic Pain or Tenderness** | | | | | | | | | | | | | | | | | | Yes | | | | | No | | Unknown | |
| **Hip or Iliac Pain or Tenderness** | | | | | | | | | | | | | | | | | | Yes | | | | | No | | Unknown | |
| **Midline Lumbar/Sacral Pain or Tenderness** | | | | | | | | | | | | | | | | | | Yes | | | | | No | | Unknown | |
| **Evidence of Genito-urinary Trauma** | | | | | | | | | | | | | | | | | | Yes | | | | | No | | Unknown | |
| **Abnormal Level of Alertness** | | | | | | | | | | | | | | | | | | Yes | | | | | No | | Unknown | |
| **Evidence of Intoxication** | | | | | | | | | | | | | | | | | | Yes | | | | | No | | Unknown | |
| **Distracting Painful Injury** | | | | | | | | | | | | | | | | | | Yes | | | | | No | | Unknown | |
| **Positive FAST Scan** | | | | | | | | | | | | | | | | | | Yes | | | | | No | | Unknown | |
| **Hematuria (gross or micro)** | | | | | | | | | | | | | | | | | | Yes | | | | | No | | Unknown | |
| **Evidence of Hypovolemia** | | | | | | | | | | | | | | | | | | Yes | | | | | No | | Unknown | |
| **Abnormal Chest X-ray** | | | | | | | | | | | | | | Yes | | | | No | | | | | Unknown | | Not Available | |
| **Abnormal Pelvic X-ray** | | | | | | | | | | | | | | Yes | | | | No | | | | | Unknown | | Not Available | |
| **Please estimate the likelihood of significant injury requiring either intervention or hospital observation/admission** | | | | | | | | | | | < 2% | | | | 2 – 10% | | | | 11 – 20% | | | | | 21 – 40% | | > 40% |
| **If estimation is < 10%, what are primary reasons for obtaining abdominal/pelvic CT (select all that apply)** | | | | | | | | | | | | | | | | | | | | | | | | | | |
|  | | Trauma protocol | | | | | | | | | | | | | | | | | | | | | | | | |
|  | | Severe mechanism of injury | | | | | | | | | | | | | | | | | | | | | | | | |
|  | | This level of risk is still important to diagnose | | | | | | | | | | | | | | | | | | | | | | | | |
|  | | Physical exam unreliable because of intoxication or altered mental status | | | | | | | | | | | | | | | | | | | | | | | | |
|  | | Medico-legal concern | | | | | | | | | | | | | | | | | | | | | | | | |
|  | | Need to diagnose all injuries even if they are not clinically significant | | | | | | | | | | | | | | | | | | | | | | | | |
|  | | Other _____________________________________________________________________________ | | | | | | | | | | | | | | | | | | | | | | | | |

**Criteria Definitions**

Terms are defined for purposes of clarity and to ensure consistent data collection.

**Date of Birth:** Based on best available information (government identification, patient response, best clinical estimate).

**Gender:** Based on direct observation.

**Race:** Based on patient response/preference, or best estimate based on observation.

**Ethnicity:** Based on patient response/preference, or best estimate based on observation.

**Patient Status:** The hemodynamic status at the time of the initial evaluation as it relates to the clinician’s ability to perform a complete and thorough evaluation of each criterion, uninterrupted by the need to provide emergent interventions.

**Coagulopathy or Anticoagulant Use:** Any impairment of normal blood clotting such as that which occurs

in hemophilia, secondary to medications (coumadin, heparin, clopidogrel, apixaban, etc.), hepatic insufficiency, and other conditions.

**Rapid Deceleration Mechanism:** As defined by the NEXUS Chest imaging rules: fall > 20 feet or motor vehicle collision > 40 miles per hour.

**Initial Oxygen Saturation (percentage):** As first measured in the emergency department.

**Initial Pulse Rate (beats/minute):** As first measured in the emergency department.

**Initial Systolic Blood Pressure (mm Hg):** As first measured in the emergency department.

**Lowest ED Oxygen saturation (percentage):** Lowest value measured in the emergency department prior to making the CT imaging decision.

**Lowest ED Pulse Rate (beats/minute):** Lowest value measured in the emergency department prior to making the CT imaging decision.

**Lowest ED Systolic Blood Pressure (mm Hg):** Lowest value measured in the emergency department prior to making the CT imaging decision.

**Initial Hemoglobin (g/dl):** As first measured in the emergency department (if obtained).

**Initial Serum Lactate (mg/dl):** As first measured in the emergency department (if obtained).

**Abdominal Pain or Tenderness:** Pain or tenderness over the abdominal area as manifest through patient complaint or elicited by direct palpation.

**Flank Pain or Tenderness:** Pain or tenderness over the lateral trunk or flank area as manifest through patient complaint or elicited by direct palpation.

**Pelvic Pain or Tenderness:** Pain or tenderness over the area of the pelvis as manifest through patient complaint or elicited by direct palpation.

**Hip or Iliac Pain or Tenderness:** Pain or tenderness over the hip or iliac area as manifest through patient complaint or elicited by direct palpation or manipulation.

**Midline Lumber/Sacral Pain or Tenderness:** Pain or tenderness over the midline lumbar or sacral spine as manifest by direct midline palpation.

**Evidence of Genito-urinary Trauma:** Including hematuria, and gross evidence of genital injury (ecchymosis, deformity, swelling, tenderness, or abrasion to the urogenital area).

**Abnormal Level of Alertness:** As evidenced by a variety of findings, including but not limited to a Glasgow coma score of 14 or less; delayed or inappropriate response to external stimuli; excessive somnolence; disorientation to person, place, time or events; inability to remember three objects at five minutes; perseverating speech; and other findings.

**Evidence of Intoxication:** Includes: a) a history of intoxication or recent intoxicating ingestion is provided by a patient or observer; b) test of bodily secretions (blood, urine, saliva, breath, etc) is positive for drugs or alcohol; c) patient has physical evidence suggesting intoxication (odor of alcohol, slurred speech, ataxia, dysmetria or other cerebellar findings), or behavior consistent with intoxication and unexplained by medical or psychiatric illness.

**Distracting Painful Injury:** Any condition thought by the clinician to produce pain sufficient to distract from a second injury, including, but not limited to long bone fracture, visceral injury requiring surgical consultation, large laceration, degloving injury, crush injury, large burns, or any injury causing acute functional impairment.

**Positive FAST Scan:** Any abnormality noted on any of the four standard FAST windows (right upper quadrant, left upper quadrant, pelvic, or cardiac).

**Hematuria (gross or micro):** As determined by direct visual observation of blood tinged urine, or urinalysis demonstrating evidence of blood (red cells or hemoglobin).

**Evidence of Hypovolemia:** Includes hypotension, tachycardia, low hemoglobin or hematocrit, falling hemoglobin or hematocrit or any other indication of decreased intravascular volume.

**Abnormal Chest X-ray:** Is considered present if there is any abnormality noted on portable chest x-ray (if obtained).

**Abnormal Pelvic X-ray:** Is considered present if there is any abnormality noted on supine pelvis x-ray (if obtained).
