# Supplementary material for: Developing a decision instrument to guide abdominal-pelvic imaging of blunt trauma patients: Methodology and protocol of the NEXUS abdominal-pelvic imaging study
Source: PLoS One. 2022 Jul 25;17(7):e0271070. doi: 10.1371/journal.pone.0271070 (PMC9312398; doi:10.1371/journal.pone.0271070)
Supplement: Attachment — Submitted filename: Response to Reviewers.docx [file pone.0271070.s002.docx]

**Response to Reviewers**

Query #1 - Journal Requirements 1 – A request to ensure that the manuscript meets PLOS ONE’s style requirements, including those for file naming.

Response #1 - We have revised the formatting and organization of the manuscript to meet PLOS ONE’s style requirements, including formatting for the main body of the manuscript, formatting of title, authors and affiliations, and formatting and numbering of references.

Query #2 - Concern that a portion of our manuscript may have been presented or published elsewhere.

Response #2 - No portion of this manuscript has been presented or published elsewhere. We did conduct pilot data collection at each of the participating centers to help us refine our protocols and ensure feasibility, and in the interest of efficiency, we plan to include this data in our final database. During this pilot work we asked clinicians to provide their estimates of the likelihood of injury for each patient, and the reason they elected to obtain imaging. This information is extrinsic to the derivation and validation of our decision instrument, but we do plan to prepare this material for separate peer-reviewed publication.

Query #3 – A request clarifying of our Data Availability statement.

Response #3 – We will provide repository information for our data at acceptance. Pending any last minute changes, we anticipate that our data will be available through the UCSF Datashare (Dash) repository at the following URL: <https://datashare.ucsf.edu/stash/dataset/doi> - pending.

Query #4 – A request to review our reference list for completeness and correctness.

Response #4 - We have reviewed and revised our reference list to ensure it is complete and correct and meets PLOS ONE’s formatting requirements. We have not changed any cited references, and our references do not included any retracted articles.

Query #5 – A reviewer request for biostatistical expert.

Response #5 – William R Mower, MD, PhD, the director of the NEXUS group and communicating author for the manuscript, holds a PhD in biomathematics and has over 30 years of experience in statistical research design. He developed the analytic plan and sample estimates for the project and will serve as the study statistician.

Query #6 - Concern that we are combining pelvic and abdominal imaging into a single decision instrument.

Response #6 - Our project will develop an instrument for combined abdominal-pelvic imaging, as this is the form of imaging that is frequently used in the evaluation of multi-trauma victims. However, the final rule will also apply to isolated abdominal imaging, or isolated pelvic imaging. This is a reflection of the fact that a patient who is designated as low risk by our instrument will have negligible risk of either abdominal or pelvic injury, and can safely be excluded from abdominal-pelvic imaging, isolated abdominal imaging, or isolated pelvic imaging.

We have included this clarification in the “General Design” section of the manuscript (lines 147 to 152 in the revised manuscript).

Query #7 – A Request to include focused assessment with sonography in trauma (FAST) in our project.

Response #7 – We recognize the importance of FAST exams in the assessment of blunt trauma patients, and have included FAST scan results as one of our candidate criteria (Supplement 1 presents our data collection instrument and contains a full listing of candidate variables). We have modified our “Introduction” to acknowledge that FAST scanning is among the imaging modalities commonly used in abdominal imaging of blunt trauma patients (lines 94 to 97 of the revised manuscript – see also Supplement 1 – Abdominal/Pelvic CT Clinical Questionnaire).

Query #8 – A reviewer is concerned that there may be cases where a clinician may want to obtain imaging regardless of the classification by the decision rule.

Response #8 - We agree that there may be instances where clinicians may want obtain imaging regardless of classification by a decision rule. Our recommendation would be to not apply the decision tool if you plan to obtain imaging regardless of classification. The goal of the tool is to help clinicians identify cases that have such low risk of injury that they may be safely excluded from imaging. Our goal is not to force imaging decisions on clinicians.

We have not changed the manuscript in response to this concern. However, it is worth noting that each of the cases described by the reviewer would be identified by our current criteria (ejected out of a car is a rapid deceleration mechanism, bilateral femoral fractures would represent distracting injuries, and intubation would exclude low risk classification for several reasons, including physiologic instability, abnormal level of alertness, and inability to complete and assessment of all criteria, which automatically excludes patients from low risk classification).

It is impossible to develop decision tools that anticipate every possible scenario, and it is possible that our final rule may occasionally misclassify patients with important injuries. If the frequency of missed injury cases cause the tool’s sensitivity to fall below our pre-specified rate of 98%, then the harm from the rule (morbidity and mortality from missed injuries) will exceed the benefits (decreased imaging with a decrease in radiation induced malignant transformation) and we would fail in our efforts to develop a decision instrument. This failure would still be informative and would provide important information for future research.

Query #9 – A request to include adrenaline use as a criterion.

Response #9 - While the use of adrenaline is likely associated with significant injuries, it is an intervention, and not a sign or symptom of injury. Adrenalin is most likely to be used in cases that exhibit hypotension, and hypotension is already included among our potential criteria.

We have mad no changes to the manuscript related to this concern.

Query 10 – Requesting a detailed analysis of the role of FAST US vs MRI in the pediatric population in the future.

Response #10 – We agree that FAST US and MRI play particularly important roles in the pediatric population, and that future assessments of these modalities would be important in defining care. However, the assessment of these modalities is beyond the scope of this project.

We have mad no change to the manuscript related to this concern.
